# Effect of bicarbonate buffer on artificial membrane permeation of drugs

**DOI:** 10.5599/admet.2603

**Published:** 2025-01-18

**Authors:** Shiori Ishida, Samuel Lee, Balint Sinko, Karl Box, Kiyohiko Sugano

**Affiliations:** 1 Molecular Pharmaceutics Lab., College of Pharmaceutical Sciences, Ritsumeikan University, 1-1-1, Noji-higashi, Kusatsu, Shiga 525-8577, Japan; 2 Pion Inc. (UK) Ltd. Forest Row Business Park, Station Road, East Sussex, RH18 5DW, United Kingdom

**Keywords:** Phosphate buffer, floating lid, lipophilic weakly acidic and basic drugs

## Abstract

**Background and purpose:**

The pH value of the small intestine is physiologically maintained by bicarbonate buffer (BCB). However, the effect of BCB on the membrane permeation of drugs has not been investigated. The purpose of this study was to investigate the effect of BCB on the passive membrane permeation of drugs.

**Experimental approach:**

The μFlux apparatus (pION Inc.) was used for permeability measurements. To avoid a pH change of BCB, a floating lid was newly developed for μFlux. The membrane filter was coated with a 10 % soybean lecithin-decane solution. The flux measurement was performed in an iso-pH condition (pH 6.5, BCB = 10 mM, buffer capacity (*β*)= 4.4 mM pH^-1^). Phosphate buffer (PPB) with the same pH and *β* was used for comparison (PPB = 8 mM).

**Key results:**

The floating lid suppressed the pH increase to less than 0.1 for 120 min. The effective permeability (*P*_e_) values of lipophilic weakly acidic and basic drugs were lower in BCB than in PPB (ketoprofen, naproxen, and propranolol). On the other hand, the *P*_e_ values in BCB and PPB were similar for unionizable drugs (caffeine and antipyrine) and hydrophilic weakly basic drugs (metoprolol and procainamide).

**Conclusion:**

Passive membrane permeation of lipophilic weakly acidic and basic drugs was slower in BCB than in PPB. This was suggested to be attributed to the slow neutralization rate of BCB, which affects the pH value adjacent to the membrane surface.

## Introduction

It is important to assess the oral absorption of a drug appropriately in drug discovery and development. The oral absorption of a drug is mainly determined by the dissolution and permeation processes in the gastrointestinal (GI) tract. These processes are affected by various physiological GI conditions such as pH, buffer species, buffer capacity (*β*), ionic strength (*I*), and bile micelle concentration [1]. The pH value of the intestinal fluid is physiologically maintained at approximately pH 6.5 by bicarbonate buffer (BCB) [2,3]. However, for practical reasons, phosphate buffer (PPB) and other buffers have been used for a variety of in vitro assays in drug discovery and drug development [4].

Biorelevant dissolution tests using BCB have been explored over the past two decades. The pH value of BCB rapidly increases as CO_2_ escapes via the air-solution interface. Previously, the CO_2_ charging methods have been used to maintain the pH value of BCB [5-8]. However, these sophisticated methods have some inherent limitations that might have limited the use of BCB in drug discovery and development [4]. Recently, the floating lid method has been introduced as a simple, robust, and versatile way to maintain the pH value of BCB [9]. The floating lid method has already been applied to compendial dissolution vessels [9], a mini-scale vessel [10], and a flow-through system [11].

BCB has a slower pH neutralization rate due to the slow CO_2_ to H_2_CO_3_ hydration/dehydration processes [12,13].



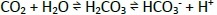

(1)


This affects the particle surface pH (pH_ps_) of ionizable drugs dissolving (and precipitating) in BCB [5,12-17]. In addition, the change of bulk phase pH (pH_bulk_) after the dissolution of ionizable drugs can also be different between BCB and PPB, even when the *β* values are set to be the same [18,19]. The p*K*_a_ values of BCB and PPB are lower and higher than 6.5, respectively (BCB: p*K*_a_ = 6.05, PPB: p*K*_a_ = 6.69, 37 °C, ionic strength (*I*) = 0.15 M) [20]. Therefore, the pH_bulk_ value is less reduced by the addition of a free weak acid or a salt of a weak base in BCB than in PPB [18,19]. These features of BCB can significantly affect the dissolution profiles of various drugs.

The dissolution profiles of free-form weakly acidic and weakly basic drugs were reported to be slower in BCB than in PPB due to the difference in pH_ps_ [5,12-15]. Recently, the dissolution profiles of salt-form drug substances were found to differ markedly between BCB and PPB due to the difference in pH_ps_ [16,21] and pH_bulk_ [19]. The crystalline precipitation of weakly acidic and basic drugs can be markedly slower in BCB than in PPB [17]. Furthermore, the dissolution profiles of many immediate-release drug products were markedly different between BCB and PPB [19,22]. The use of BCB in dissolution tests was suggested to be critically important for the assessment of bioequivalence in generic drug development [11,23-25]. However, to the best of our knowledge, the effect of BCB on the membrane permeation process has not been investigated.

**Figure 1.**
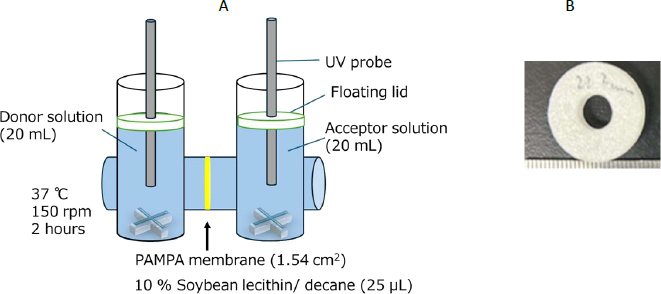
Flux apparatus with floating lids (A) and the picture of the floating lid (B)

The purpose of the present study was to investigate the effect of BCB and PPB on the passive membrane permeation of drugs. In this study, three weakly acidic drugs, three weakly basic drugs, and two neutral drugs were used as model drugs (Table 1). The μFlux apparatus (pION Inc.) was used for permeability measurements. A floating lid was newly developed for μFlux (Figure 1) [9]. BCB condition was set to be relevant to the human small intestine (pH 6.5, BCB = 10 mM, *I* = 0.14 M) [2,3]. The μFlux experiment used an artificial membrane composed of phospholipids and an organic solvent, the same as in the parallel artificial membrane permeabilization assay (PAMPA). PAMPA has been widely used to assess the passive membrane permeation of drugs in drug discovery and development [26,27].

**Table 1.** Physicochemical properties of model drugs

Drug name	MW	log *P*_oct_^a^	p*K*_a_^a^	log *D*_oct_ (pH 6.5)^d^	*D*_mono_ / 10^-6^ cm^2^ s^-1e^	UV detection, nm
Antipyrine	188	0.56	-	0.56	9.23	280
Caffeine	194	-0.01	-	-0.01	9.10	290
Furosemide	331	2.56	3.53 (A)^b^	-0.41	7.15	330
Ketoprofen	254	3.16	4.00 (A)^b^	0.66	8.05	280
Metoprolol	267	1.95	9.24 (B)^c^	-0.79	7.87	276
Naproxen	230	3.24	4.14 (A)^b^	0.88	8.42	320
Procainamide	235	1.49	9.01 (B)^c^	-1.02	8.34	310
Propranolol	259	3.48	9.16 (B)^b^	0.82	7.98	320

^a^25 °C [20];

^b^37 °C, *I* = 0.15 M;

^c^Converted to the value at 37 °C from the value measured at 25 °C, *I* = 0.15 M [28].

^d^Octanol - water distribution coefficient at pH 6.5. Calculated from log *P*_oct_ and p*K*_a_.

^e^Diffusion coefficient of monomer drug in water at 37 °C (*D*_mono_ = 9.9×10^-5^ MW^-0.453^ cm^2^ s^-1^)[29]

## Experimental

### Material

The floating lid for μFlux was made of polystyrene foam (outer diameter: 22.2 mm, inner diameter: 7.0 mm, thickness: 3 mm). A laser cutter was used to precisely cut polystyrene foam with an accuracy of 0.1 mm. Soybean lecithin was provided by Tsuji Oil Mills Co., Ltd (Mie, Japan) (SLP-White, phosphatidylcholine (24 to 32 %), phosphatidylethanolamine (20 to 28 %), phosphatidylinositol (12 to 20 %), phosphatidic acid (8 to 15 %), and lysophosphatidylcholines (1 to 5 %) (based on the product information provided by the manufacturer)). Antipyrine, ketoprofen, naproxen, propranolol, NaH_2_PO_4_·2H_2_O, NaHCO_3_, NaCl, 8 M NaOH and 6 M HCl were purchased from Wako Pure Chemical Industries, Ltd. (Osaka, Japan). Caffeine, furosemide, and metoprolol were purchased from Tokyo Chemical Industry (Osaka, Japan). Procainamide was purchased from Abcam Limited (Cambridge, MA, USA).

### Methods

#### pH maintenance in μFlux apparatus with floating lid

The membrane filter of the μFlux apparatus (pION Inc., Billerica, MA 01821, USA) was coated with 25 μL of a 10 % soybean lecithin-decane solution [30]. A NaHCO_3_ solution (19.6 ml, 10.1 mM, NaCl 0.13 M) was added to both the donor and acceptor chambers. An HCl solution (0.4 ml, 0.17 M) was added to adjust the pH value to pH 6.5 (final conditions: BCB = 10 mM, *β* = 4.4 mM pH^-1^, *I* = 0.14 M). The *β* value was calculated by the van Slyke equation [31] using the second pK_a_ values of buffer species, assuming the activity coefficient is 1. The temperature was maintained at 37 °C. The paddle rotation speed was set to 150 rpm. The solution surface was covered by a floating lid (Figure 1). The bulk phase pH value was measured at each time point using a Gel-filled pH sensor cartridge 300-P-C (HORIBA Advanced Techno, Co., Ltd., Kyoto, Japan). The pH maintenance test was performed in triplicate.

#### Flux measurement

The flux measurement was performed in an iso-pH condition (pH 6.5 for both donor and acceptor solutions). The lipid membrane was prepared as described above. A NaHCO_3_ solution (98 ml, 10.1 mM, NaCl 0.13 M) was added to a 100 mL beaker. An HCl solution (2 ml, 0.17 M) was added to adjust the pH value to pH 6.5 (final conditions: BCB = 10 mM (*β* = 4.4 mM pH^-1^), *I* = 0.14 M). Each drug was dissolved, and pH was re-adjusted to pH 6.5 by adding a small amount of 1 M NaOH or 1 M HCl. The initial concentration of each drug in the donor solution (*C*_D, init_) was set so that the drug concentration in the acceptor solution could be detected (Table 2). It was visually confirmed that the added drug was completely dissolved. A floating lid for the 100 mL beaker was used during the above procedures (covering >95 % of the air-solution interface). The drug solution (20 mL) was added to the donor chamber and the blank solution (20 mL) was added to the acceptor chamber. The solution surface was covered by a floating lid for μFlux. PPB was used for comparison (pH 6.5, PPB = 8 mM (*β* = 4.4 mM pH^-1^), *I* = 0.14 M). The temperature was maintained at 37 °C. The paddle rotation speed was set to 150 rpm. The drug concentrations in the donor and acceptor solutions were determined by a UV probe (2 and 20 mm apertures, respectively) (Table 1). The initial and final bulk phase pH values were measured as described above. The flux value (*J*) was calculated from the slope of the drug concentration-time curve in the acceptor solution from 30 to 120 min. The effective permeability (*P*_e_) value was calculated from the flux value and the final donor concentration (*C*_D, final_) as *P*_e_ = *J* / *C*_D, final_. The flux measurement was performed in triplicate.

**Table 2.** Flux and permeability data^a^

Drug name (*C*_D, init_ / mg mL^-1^)	Buffer	*J /* μg cm^-2^ min^-1^	*P*_e_ / 10^-6^ cm s^-1^	Final pH (at 120 min)	
				Donor	Acceptor
Antipyrine	PPB	1.27 ± 0.04	2.27 ± 0.06	6.50 ± 0.02	6.50 ± 0.01
(5.0 mg/mL)	BCB	1.17 ± 0.03	2.14 ± 0.07	6.57 ± 0.03	6.58 ± 0.02
Caffeine	PPB	2.05 ± 0.09	3.54 ± 0.19	6.53 ± 0.02	6.48 ± 0.00
(10 mg/mL)	BCB	1.97 ± 0.09	3.39 ± 0.12	6.55 ± 0.02	6.54 ± 0.01
Ketoprofen	PPB	0.465 ± 0.016	7.33 ± 0.28	6.46 ± 0.02	6.47 ± 0.00
(1.0 mg/mL)	BCB	0.280 ± 0.017	6.01 ± 0.44	6.59 ± 0.01	6.46 ± 0.01
Metoprolol	PPB	0.889 ± 0.062	14.8 ± 1.0	6.46 ± 0.02	6.45 ± 0.01
(1.0 mg/mL)	BCB	0.902 ± 0.044	14.4 ± 0.7	6.62 ± 0.03	6.59 ± 0.00
Naproxen	PPB	0.888 ± 0.030	14.7 ± 0.5	6.50 ± 0.02	6.47 ± 0.02
(1.0 mg/mL)	BCB	0.637 ± 0.005	10.8 ± 0.9	6.62 ± 0.01	6.63 ± 0.01
Procainamide	PPB	0.594 ± 0.013	1.05 ± 0.02	6.46 ± 0.03	6.46 ± 0.00
(10 mg/mL)	BCB	0.594 ± 0.014	1.05 ± 0.03	6.64 ± 0.04	6.62 ± 0.00
Propranolol	PPB	1.22 ± 0.03	43.5 ± 0.9	6.47 ± 0.03	6.46 ± 0.01
(0.5 mg/mL)	BCB	0.956 ± 0.030	33.3 ± 1.0	6.56 ± 0.01	6.60 ± 0.01

^a^Mean ± S.D., *N* = 3. Furosemide data was not shown as it was not detected in the acceptor solution.

## Results and discussion

**Figure 2.**
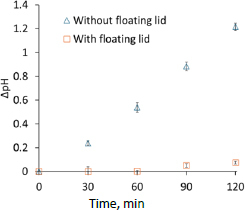
pH change of donor solution with or without floating lid. Mean ± S.D., *N* = 3.

First, the pH maintenance performance of the floating lid was evaluated for μFlux (Figure 1). Without the floating lid, the pH value of BCB was rapidly increased by the loss of CO_2_ (Figure 2). The floating lid suppressed the pH increase completely until 60 min and to less than 0.1 for 120 min. As the surface-to-volume ratio becomes smaller, such as in the case of μFlux, it becomes more challenging to maintain the pH value by a floating lid. In this study, a precise laser cutter was used to produce the floating lid for μFlux. The pH value was well maintained by the floating lid method, suggesting that this method can be used for further flux measurements.

**Figure 3.**
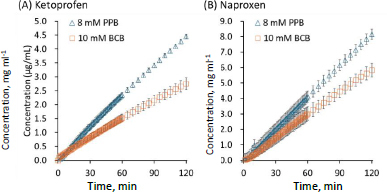
Concentration time profiles of weakly acidic drugs in the acceptor chamber. Mean ± S.D., *N* = 3.

**Figure 4.**
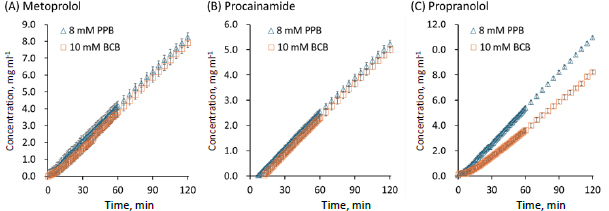
Concentration time profiles of weakly basic drugs in the acceptor chamber. Mean ± S.D., *N* = 3.

**Figure 5.**
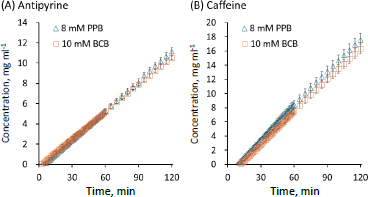
Concentration time profiles of unionizable drugs in the acceptor chamber. Mean ± S.D., *N* = 3.

In the flux measurements, the initial values of pH_bulk_ were 6.5 ± 0.0 and the final values of pH_bulk_ at 120 min were 6.5 ± 0.1 in all cases (Table 2). The *P*_e_ values of lipophilic weakly acidic drugs (ketoprofen and naproxen, log *D*_oct,pH 6.5_ >0) were lower in BCB than in PPB (Figure 3). The *P*_e_ value of a lipophilic weakly basic drug (propranolol) was also lower in BCB than in PPB (Figure 4C). On the other hand, the *P*_e_ values of unionizable drugs (caffeine and antipyrine) in BCB and PPB were similar (Figure 5). The *P*_e_ values of hydrophilic weakly basic drugs (metoprolol and procainamide, log *D*_oct,pH 6.5_ < 0) in BCB and PPB were also similar (Figures 4A and 4B). The flux measurement was also performed for a hydrophilic weakly acid drug (furosemide). However, the drug was not detected in the acceptor solution even when the donor concentration was increased to its solubility limit (data not shown).

In this study, it was found for the first time that the effective permeability of lipophilic weakly acidic and basic drugs (ketoprofen, naproxen, propranolol) (log *D*_oct,pH 6.5_ >0) was slower in BCB than in PPB. *P*_e_ is determined as the sum of the reciprocals of the unstirred water layer (UWL) permeability (*P*_UWL_) adjacent to the membrane and the membrane permeability (*P*_m_) (1/ *P*_e_ = 1/ *P*_UWL_ + 1/ *P*_m_). *P*_UWL_ can be calculated from the diffusion coefficient of a monomer drug (*D*_mono_) and the thickness of the UWL (*h*_UWL_) as *P*_UWL_ = *D*_mono_ / *h*_UWL_. The *h*_UWL_ value at 150 rpm was calculated to be 164 μm by the rpm-to-*h*_UWL_ converter provided by pION Inc. *D*_mono_ can be estimated from MW (Table 1). For all model drugs, *P*_UWL_ was calculated to be greater than 4×10^-4^ cm s^-1^. Therefore, *P*_e_ was lower than *P*_UWL_ and the membrane permeation was the rate-limiting process in all cases (*P*_e_ ≈ *P*_m_). Therefore, the difference in *P*_e_ between BCB and PPB was suggested to be attributed to their different effects on the pH value adjacent to the membrane surface (pH_ms_).

**Figure 6.**
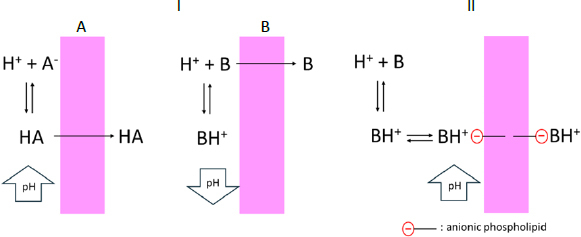
Permeation of drugs as an unionized form (I) or an ion-pair (II) across the lipid membrane. (I-A) Permeation of unionized weakly acidic drug molecules (HA) removes H^+^ and increases pH adjacent to the membrane surface on the donor side (pH_ms_). (I-B) Permeation of unionized weakly basic drug molecules (B) increases H^+^ and decreases pH_ms_. (II) Permeation of ionized (protonated) weakly basic drug molecules removes H^+^ and increases pH_ms_.

According to the pH partition theory, only unionized species can permeate a lipid membrane (Figure 6). Therefore, the unionized fraction (*f*_0_) of a drug at the membrane surface affects *P*_e_. In the case of a lipophilic weakly acidic drug, the membrane permeation of unionized species (HA) removes proton (H^+^) from the solution on the donor side, increases pH_ms_, and subsequently decreases *f*_0_ and *P*_e_. Because the pH neutralization rate of BCB is slower than that of PPB, *P*_e_ in BCB becomes lower than that in PPB. The same discussion can also be applied to lipophilic weakly basic drugs.

In the case of hydrophilic weakly basic drugs (metoprolol and procainamide), the *P_e_* values in BCB and PPB were similar. The *P*_e_ values of these drugs were higher than expected from their low log *D*_oct,pH 6.5_ values (< -1), suggesting that their membrane permeation cannot be elucidated solely by the pH partition theory. Previously, the passive membrane permeation of ionized hydrophilic weakly basic drug molecules has been reported to occur via ion-pair transport with an anionic phospholipid in the membrane, such as phosphatidylinositol (Figure 6II) [32,33]. In this case, the *P*_e_ values were reported to be less pH sensitive and do not follow the pH partitioning theory in a pH range where ion-pair transport becomes predominant [32]. Theoretically, ion-pair transport would depend on the fraction of cationic species (*f*_+_). The permeation of the protonated species of a weakly basic drug (BH^+^) as an ion-pair with an anionic phospholipid may increase the pH_ms_ value. However, the *f*_+_ values of these drugs are little affected by a difference in pH_ms_ (*f*_+_ > 0.99), resulting in similar *P*_e_ values between BCB and PPB.

The theoretical calculation of pH_ms_ is of great interest and is currently under investigation. At this moment, it is unknown whether the result of this study in the PAMPA membrane would be relevant to in vitro cellular models and/or in vivo. The microclimate pH near the epithelial membrane is well controlled in vivo [34]. Further investigation is required to clarify this point. In *β* calculation, when the operational electrode pH was converted to [H^+^] (*I* = 0.15 M) by the Avdeef-Bucher four-parameter equation [20] and all p*K*_a_ values (BCB: 6.05; 9.79, PPB: 1.94; 6.69; 11.61 (*I* = 0.15 M, 37 °C)) [20] are used for the 3 p*K*_a_ van Slyke equation [31], the *β* values were calculated to be 4.9 and 4.1 for BCB (10 mM) and PPB (8.0 mM), respectively. Previously, the *β* values of BCB 10 mM and PPB 8 mM at pH 6.5 were experimentally determined to be 4.6 and 4.5 mmol L^-1^ pH^-1^, respectively [19]. The higher the *β* value, the lower the decrease in drug permeation due to pH_ms_ change, as is the case for pH_ps_ [35-37]. The effect of this difference on *P*_e_ may be small but could be a possible factor in addition to the slow rate of carbon dioxide hydration.

## Conclusions

In conclusion, the floating lid method enabled the use of BCB in μFlux. It was found for the first time that the membrane permeation of lipophilic weakly acidic and basic drugs can be slower in BCB than in PPB. It was suggested that this effect was attributed to the slow pH neutralization rate of BCB at the membrane surface.
